# A132 A CASE OF COLONIC MALAKOPLAKIA PRESENTING AS A LARGE CECAL MASS : NOT EVERY POLYP HAS TO LOOK LIKE A NAIL

**DOI:** 10.1093/jcag/gwac036.132

**Published:** 2023-03-07

**Authors:** S X Jiang, W Xiong, N Shahidi

**Affiliations:** 1 Medicine; 2 Pathology; 3 Gastroenterology, University of British Columbia, Vancouver, Canada

## Abstract

**Background:**

Malakoplakia is a rare inflammatory condition, commonly affecting the genitourinary system of immunocompromised patients. Gastrointestinal malakoplakia has been reported in the literature, with previous endoscopic findings mimicking mass lesions.

**Purpose:**

To describe a case of colonic malakoplakia after referral for endoscopic management of a large non-pedunculated colorectal polyp.

**Method:**

Case report and review of the literature.

**Result(s):**

**Case Report**

An 80-year-old male with chronic kidney disease and remote renal transplant on immunosuppressive therapy (mycophenolate mofetil, tacrolimus, prednisone) was referred for endoscopic resection for a large non-pedunculated colorectal polyp in the cecum. Original presenting symptoms included chronic diarrhea, iron deficiency anemia, and fecal immunochemical test (FIT) positivity. A repeat colonoscopy demonstrated a 40mm Paris 0-IIA plaque-like lesion in the cecum with optical features not in keeping with adenomatous or serrated histopathology. Biopsies were performed with histopathology demonstrating normal colonic mucosa with confluent sheets of histiocytes with concentrically layered cytoplasmic inclusions, in keeping with malakoplakia.

**Literature review**

Malakoplakia, Greek for “soft plaque”, is a rare inflammatory condition characterized by impaired dysfunction in macrophages leading to the accumulation of incompletely degraded bacteria in phagolysosomes. Histologically, this appears as concentrically layered cytoplasmic inclusions, comprising the pathognomonic Michaelis-Gutmann bodies. Many bacterial organisms have been implicated in the development of malakoplakia, with *E. coli* being the most common; specifically in immunocompromised patients, whether from immunosuppressive medications, immunodeficiency syndromes, or clinical conditions precluding effective immune function. Malakoplakia commonly presents as a mass-like lesion and has been found in all organs, most commonly in the genitourinary system. Diagnosis is made by biopsy and allows for appropriate treatment, which is most commonly a reduction in immunosuppressive therapy and antibiotic therapy.

**Image:**

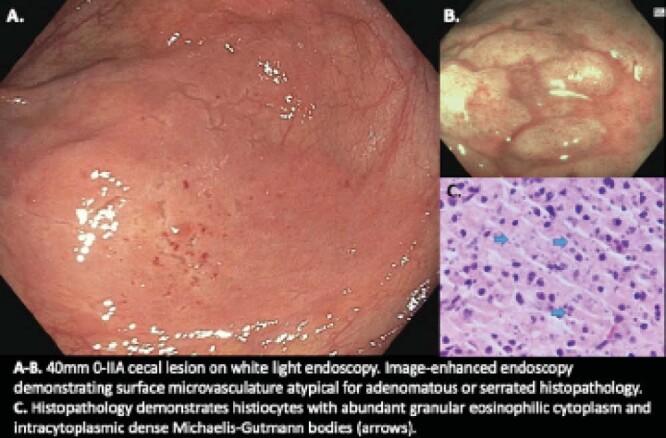

**Conclusion(s):**

Malakoplakia is rare but should be considered when a lesion with atypical optical evaluation features is found in immunocompromised individuals or those with recurrent infections.

**Please acknowledge all funding agencies by checking the applicable boxes below:**

None

**Disclosure of Interest:**

None Declared

